# Pre-operative intravitreal dexamethasone implant in patients with refractory diabetic macular edema undergoing cataract surgery

**DOI:** 10.1038/s41598-020-62561-3

**Published:** 2020-03-26

**Authors:** Stamatina A. Kabanarou, Tina Xirou, Eirini Boutouri, Ilias Gkizis, Dimitrios Vasilias, Georgios Bontzos, Irini Chatziralli

**Affiliations:** 1 Department of Ophthalmology, Korgialenio-Benakio Hospital, Athens, Greece; 2 2nd Department of Ophthalmology, University of Athens, Athens, Greece

**Keywords:** Medical research, Signs and symptoms

## Abstract

To examine preoperative use of intravitreal dexamethasone implant in patients with refractory diabetic macular edema (DME) undergoing cataract surgery. Participants in this study were 17 patients with DME refractory to previous treatment with anti-vascular endothelial growth factor agents or dexamethasone implant, and co-existent cataract. All participants received intravitreal dexamethasone implant at baseline and underwent phacoemulsification within one month after its insertion. Best-corrected visual acuity (BCVA) and central subfield thickness (CST) changes between baseline, time of cataract surgery and postoperative months 1, 2 and 3 were evaluated. At month 1 after surgery, BCVA improved significantly from 42.3 ± 9.6 to 58.7 ± 11.9 letters compared to baseline (p < 0.001) and the improvement was sustained at month 2 and month 3 postoperatively. One month postoperatively, CST improved significantly compared to baseline (p < 0.001) and the improvement was sustained at month 2 (p < 0.001), while at month 3 CST started to increase, but remained significantly lower than baseline (p = 0.003). At month 3 postoperatively, 35.3% of patients presented recurrence of ME. Patients with refractory DME and cataract can safely undergo phacoemulsification when dexamethasone implant is inserted one month prior to surgery to ensure adequate control of postoperative inflammation and prevent deterioration of ME.

## Introduction

Diabetic macular edema (DME) is a common complication of diabetes mellitus, affecting about 20% of patients with diabetic retinopathy, and can lead to visual loss if left untreated^1,2^. Patients with diabetes mellitus seem to develop cataract more frequently and earlier compared to non-diabetic population, because of the inherent metabolic condition of the disease^3–5^. It is worthy to note that cataract surgery in diabetic patients has been associated with higher risk of complications, including postoperative macular edema (Irvine-Gass syndrome) or worsening of pre-existing DME, especially in the presence of unstable diabetic retinopathy^6–12^. In fact, it has been reported that about 22% of diabetic patients, who undergo cataract surgery, develop macular edema^7^.

Previous studies have shown that intravitreal injection of anti-vascular endothelial growth factor (anti-VEGF) agents or triamcinolone pre-operatively, post-operatively or at the time of phacoemulsification may reduce the development of postoperative DME, although rapid recurrence of macular edema occurred and in case of triamcinolone the risk of intraocular pressure (IOP) increase may also exist^13–22^.

Intravitreal dexamethasone implant (Ozurdex, Allergan) is a biodegradable corticosteroid implant, which provides sustained-release of 700 μg dexamethasone into the vitreous for up to 6 months^23^. It has been successfully applied for the treatment of DME, retinal vein occlusion and non-infectious posterior uveitis^24–26^. There are few studies using intravitreal dexamethasone implant to control postoperative macular edema in patients affected by DME, showing promising results^27–32^. Most studies reported the use of implant at the same time of cataract operation. Specifically, Panozzo *et al*. have found that DME occurred in 1 out of 19 patients with DME and cataract one month after surgery, and in 74% of patients at month 4–5 postoperatively, in patients who were treated with phacoemulsification and dexamethasone implant at the time of cataract operation^29^. Malclès *et al*. in their retrospective study described the application of dexamethasone implant one month before cataract surgery^32^.

In light of the above, the purpose of this study was to examine the anatomical and functional results in patients with refractory DME and cataract, who received intravitreal dexamethasone implant and subsequently underwent phacoemulsification one month thereafter. The rationale of the use of intravitreal dexamethasone one month prior to cataract surgery is to benefit from its peak of action, so as to prevent deterioration of DME more effectively or even improve macular edema postoperatively.

## Results

Table 1 shows the demographic and clinical characteristics of our study sample. The mean age of patients was 72.3 ± 7.1 years. 8 patients (47.1%) were male and 9 (52.9%) female. The mean HbA1c was 7.3 ± 0.7% and patients had a mean duration of diabetes mellitus of 14.1 ± 5.3 years. 14 patients (82.4%) presented non-proliferative diabetic retinopathy and 3 patients (17.6%) non-active proliferative diabetic retinopathy previously treated with pan-retinal photocoagulation. Hypertension was present in 14 patients (82.4%) and hyperlipidaemia in 9 patients (52.9%). All patients had received previously at least 3 intravitreal ranibizumab injections with a mean number of 6.9 ± 5.8 injections (range: 3–25 injections), while 6 patients (35.3%) were previously treated with one intravitreal dexamethasone implant (at least 6 months before recruitment for this study) and 3 patients (17.6%) with macular focal/grid laser. Regarding the lens status, 10 patients (58.8%) had nuclear cataract, 3 patients (17.6%) cortical cataract and 4 patients (23.6%) posterior subcapsular cataract.

**Table 1:** Demographic and clinical characteristics of our study sample (n = 17 eyes) at baseline (at intravitreal dexamethasone implant insertion).

**Age (years, mean ± standard deviation)**	72.3 ± 7.1
**Gender (N, %)**	
*Male*	8 (47.1)
*Female*	9 (52.9)
**HbA1c (%, mean** ± **standard deviation)**	7.3 ± 0.7
**Duration of diabetes mellitus (years, mean** ± **standard deviation)**	14.1 ± 5.3
**Diabetic retinopathy status (N, %)**	
*Non-proliferative*	14 (82.4)
*Proliferative (quiescent)*	3 (17.6)
**Hypertension (N, %)**	14 (82.4)
**Hyperlipidaemia (N, %)**	9 (52.9)
**Lens status (N, %)**	
*Nuclear cataract*	10 (58.8%)
*Cortical cataract*	3 (17.6%)
*Posterior subcapsular cataract*	4 (23.6%)
**Previous treatment (N, %)**	
*Intravitreal ranibizumab*	17 (100)
*Intravitreal dexamethasone implant*	6 (35.3)
*Focal/grid laser*	3 (17.6)
**Previous intravitreal injections (mean** ± **standard deviation, range)**	6.9 ± 5.8 (3–25)
**Best corrected visual acuity (ETDRS letters, mean** ± **standard deviation)**	42.3 ± 9.6
**Central subfield thickness (μm, mean** ± **standard deviation)**	479.3 ± 89.7
**Intraocular pressure (mmHg, mean** ± **standard deviation)**	14.9 ± 1.8

**Figure 1:**
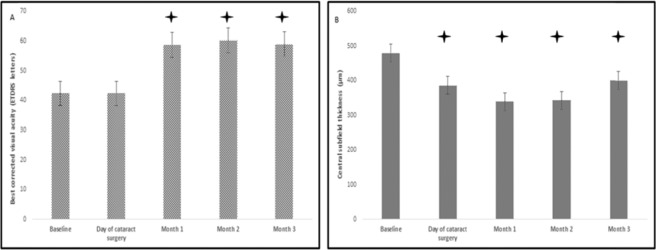
(**A**) Evolution of best corrected visual acuity over time. The bars represent mean±standard error. Stars show statistical significance (p < 0.05) compared to baseline. (**B**) Evolution of central subfield thickness over time in patients with diabetic macular edema and concurrent cataract, treated with intravitreal dexamethasone one month prior to cataract surgery. The bars represent mean±standard error. Stars show statistical significance (p < 0.05) compared to baseline.

At baseline, the mean BCVA was 42.3 ± 9.6 letters and remained stable until cataract surgery (42.3 ± 9.4 letters). At month 1 after cataract surgery, BCVA improved significantly to 58.7 ± 11.9 letters compared to baseline (p < 0.001) and the improvement was sustained at postoperative month 2 (60.2 ± 12.1 letters, p < 0.001) and month 3 (58.9 ± 11.8 letters, p < 0.001) compared to baseline. The evolution of BCVA over time is depicted in Fig. 1A.

At baseline, the mean CST was 479.3 ± 89.7 μm and improved significantly to 386.2 ± 76.9 μm one month after injection, at the day of cataract surgery (p < 0.001). At month 1 after cataract surgery, CST improved significantly to 339.1 ± 96.7 μm compared to baseline (p < 0.001) and the improvement was sustained at postoperative month 2 (342.3 ± 95.2 μm, p < 0.001 compared to baseline), while at month 3 postoperatively CST started to increase but remained significantly lower than baseline (400.8 ± 102.9 μm, p = 0.003). The evolution of CST over time is depicted in Fig. 1B. At months 1 and 2 postoperatively, all patients showed improvement or even total absorption of macular edema. Specifically, 8 out of 17 patients (47.1%) at month 1 postoperatively and 7 out of 17 patients (41.2%) at month 2 postoperatively presented total resolution of macular edema (CST < 320 μm). At postoperative month 3 (4 months after intravitreal dexamethasone implant insertion), 5 out of 17 patients (29.4%) had resolution of macular edema, while 6 out of 17 patients (35.3%) presented recurrence of macular edema, as it is detected on SD-OCT. It is worthy to note that only one out of 6 eyes with recurrent macular edema had received previous treatment with dexamethasone implant.

As far as the complications are concerned, no serious systemic side effects were reported from any of the patients in the study. No thromboembolic or cardiovascular events were mentioned. In addition, there was no inflammatory reaction, endophthalmitis or retinal tears. One patient (5.9%) had raised IOP (>35 mmHg) two months postoperatively, which was controlled with topical anti-hypertensive agents.

## Discussion

The principal message of this prospective study was that intravitreal dexamethasone implant used one month prior to cataract surgery was an effective and safe strategy to deal with patients with refractory DME and concurrent cataract. Specifically, there was significant improvement in BCVA and CST postoperatively until the 3-month follow-up after surgery, without serious ocular complications, suggesting that such patients benefit from cataract surgery and did not experience deterioration of DME secondary to potential postoperative inflammation.

It is worthy to mention that CST decreased significantly one month after the implant insertion (just before scheduled cataract operation), although visual acuity remained the same, suggesting that the presence of cataract was indeed a sight limiting factor that did not allow the anatomical improvement in the fovea to be reflected in the functional outcome. Moreover, one month after cataract surgery (two months after the implant insertion) CST improved significantly further compared to baseline and to preoperative measurement, indicating that the effect of the dexamethasone not only halted worsening of macular edema due to the inflammatory process that evolves intraoperatively and at the immediate post-cataract period, but also facilitated further resolution of the edema. Furthermore, since VA improved significantly only after cataract surgery compared to baseline, we suggested that patients with DME and advanced cataract do benefit from cataract surgery, even in cases with a history of resistant DME to either anti-VEGF or dexamethasone implant.

Previous studies have shown that DME may worsen after cataract surgery^8–12^, while macular edema may also occur postoperatively in patients without previous macular impairment^12^. Intravitreal dexamethasone implant has been shown to have similar functional results compared to anti-VEGF agents in patients with DME, but with better anatomical response and fewer injections. Specifically, in a recent meta-analysis by He *et al*., intravitreal dexamethasone implant presented superior anatomic outcomes in 6 months with mean CST reduction of about 87 μm compared to anti-VEGF treatment (mean CST reduction of about 34 μm), similar to our results showing about 80 μm reduction of CST at month 3 postoperatively and 4 months after intravitreal dexamethasone implant^33^. In addition, the benefit of intravitreal dexamethasone implant in DME reached its peak at 4–8 weeks after insertion^23^, being a reasonable option in patients with DME and concurrent cataract, since it covers the time interval of presumed postoperative inflammatory reaction, apart from facilitating the resolution of pre-existing macular edema^29^.

Following the evolution of CST in our study, we have noticed that the maximum action of dexamethasone implant occurred at month 1 postoperatively, about 60 days after dexamethasone implant insertion. This can be explained based on the pharmacodynamics of the drug, showing that the mean reduction in CST reaches its highest point when dexamethasone reaches its highest concentration, happening about 30–60 days after implant^23^. This was also the time where the maximum increase in VA occurred, which can be attributed both to the cataract extraction and to the thinner CST, which can justify our initial hypothesis to insert the implant one month before surgery to maximize its protective role on macular edema. At month 3 postoperatively (four months after the implant injection), CST started to increase as the effect of the drug wears off, which is in accordance with the duration of action of the implant^23,24,34^. At that time recurrence of macular edema occurred in 35% of cases suggesting that re-insertion of dexamethasone implant may be needed. Accordingly, BCVA was significantly improved at month 1 after surgery and remained stable till the end of the follow-up, suggesting that patients with sight-limiting cataract may benefit from surgery, even in the presence of DME.

Regarding the optimal time of intravitreal dexamethasone implant insertion in patients with DME and cataract, there are various approaches. Previous studies have used dexamethasone implant either at the beginning or immediately after surgery in an attempt to prevent or control postoperative macular edema^27–31,35^. Agarwal *et al*. used intravitreal dexamethasone implant in 18 eyes with DME and cataract, at the beginning of phacoemulsification, reporting 15–18 letters gain in visual acuity compared to stable visual acuity in controls associated with a decrease in CMT^35^, while Sze *et al*. also showed improvement in BCVA and CST using dexamethasone implant just before initiation of cataract surgery, but about 10% of patients required further treatment due to recurrence of macular edema in about 21 weeks post-operation^28^. Other authors preferred to insert the intravitreal dexamethasone implant at the end of the surgery, when potential intraoperative complications were overcome and a better visualization of the implant in the vitreous was possible, also demonstrated good anatomical and functional outcomes^27,29–31^. In our study, however, about 35% of patients presented recurrent macular edema 3 months postoperatively, suggesting that re-insertion of dexamethasone implant may be needed. The discrepancy of our results and those of previous studies could be potentially attributed to the variation of population, to the difference in macular edema specific features and probably to the difference in the timing of implant insertion.

An interesting point that should be taken into account pertains to the distinction between DME and postoperative macular edema (Irvine-Gass syndrome), which is challenging in such patients. However, the incidence of Irvine-Gass syndrome after modern phacoemulsification is very low, ranging between 0.1–2.4%, although it is reported to be much higher in diabetic patients reaching 20%^7,12,36^. Moreover, in the majority of cases, Irvine-Gass syndrome occur between 4–10 weeks postoperatively, which is shorter than the mean time of recurrence of macular edema both in our study and in previous reports^37,38^, while other studies supported that the inflammatory signal process after cataract surgery is believed to be short-lived and macular edema may develop in as little as 1 month after surgery^30,39^. By inserting the implant one month prior to cataract surgery, the peak action of the drug (30–60 days) and duration of action of the drug (roughly 4 months) can theoretically control more effectively both diabetic macular edema and Irvine-Gass syndrome, especially in cases where either or both of them occurred within the first month after cataract surgery, as previously reported^7,30,39^.

This study reports the pre-operative use of intravitreal dexamethasone implant within one month prior to surgery to halt progression of macular edema postoperatively, showing improvement in BCVA and CST at the 3-month follow-up after surgery. Although we acknowledge the fact that an additional surgical procedure is needed if dexamethasone implant was inserted one month pre-operatively, a better timing for control of postoperative inflammation is ensured. A safer implant injection was also guaranteed, since insertion either at the beginning or at the end of the cataract procedure has potential risks. Specifically, if the implant is inserted at the beginning of cataract surgery, the surgeon has to remove the implant in case of posterior capsule rupture, to eliminate the possibility of its migration into the anterior chamber and its damaging effect on the cornea^30^. On the other hand, if the implant is inserted at the end of cataract surgery, there might be a risk of potential hazardous manipulations to the globe and to the anterior chamber stability that can endanger cataract surgery outcome^28^.

Potential limitations of the study were the small sample size and the lack of control group, but one should take into consideration ethical issues to recruit patients as controls and perform no treatment to DME. In addition, we did not included data on the parameters of phacoemulsification, which could help to estimate a correlation between the amount of energy used during surgery and postoperative retinal thickness. Nevertheless, the strengths of this study include the prospective design and the use of SD-OCT to detect macular edema.

In conclusion, this study demonstrated promising anatomical and functional results in cataract patients with DME, treated with intravitreal dexamethasone implant one month prior to scheduled cataract extraction, although it should be taken into account that this is a study without control group. Further studies with larger study sample and longer follow-up are needed to confirm further the pre-operative use of intravitreal dexamethasone implant in the treatment of DME patients undergoing cataract surgery.

## Methods

This is a prospective study, which was conducted at the Korgialenio Benakio Hospital, Athens, Greece. Participants in the study were 17 patients (17 eyes) with type 2 diabetes mellitus and DME refractory to previous treatment with anti-VEGF agents or dexamethasone implant, who had co-existent cataract. Specifically, all participants had refractory DME to the affected eye (central subfield thickness – CST > 320 μm), which had been previously treated with at least 3 intravitreal ranibizumab injections or one dexamethasone implant, showing no response, defined as reduction in CST less than 50 μm. Patients with macular edema secondary to causes other than diabetes mellitus, patients with active proliferative diabetic retinopathy (PDR), ocular inflammation and advanced glaucoma were excluded from the study. The study was in accordance with the Tenets of Helsinki Declaration and was approved by the institutional review board of Red Cross “Korgialenio-Benakio” Hospital. Written informed consent was obtained from all participants before enrollment to the study.

All participants underwent a complete ophthalmic examination at baseline (before intravitreal dexamethasone implant insertion), including best corrected visual acuity (BCVA) measurement by means of ETDRS letters chart, slit-lamp examination, IOP measurement, dilated fundoscopy and spectral-domain optical coherence tomography to measure CST using Spectralis (SD-OCT; Spectralis HRA-OCT, Heidelberg Engineering, Heidelberg, Germany).

At the diagnosis of DME (baseline), all participants received intravitreal dexamethasone implant, which was performed under sterile protocol, which included the use of 5% povidone-iodine solution, topical anesthesia, eyelid-speculum application, intravitreal injection of 0.7 mg dexamethasone implant via pars plana in the infero-temporal quadrant at 4 mm from the limbus, followed by post-injection topical antibiotic eye-drops medication.

All patients underwent a standard uncomplicated phacoemulsification with posterior chamber intraocular lens (IOL) implantation under topical anesthesia, within one month after dexamethasone implant insertion, where BCVA, IOP and CST were also measured. Thereafter, we assessed BCVA, IOP and CST in all patients monthly postoperatively for at least 3 months. The main outcomes were BCVA and CST changes between baseline (dexamethasone implant insertion), time of cataract surgery and months 1, 2 and 3 post-cataract extraction. Safety was evaluated by documenting intraoperative or postoperative complications, changes in IOP, the use of IOP-lowering agents and any patient-reported or investigator-reported adverse events.

Statistical analysis was performed using SPSS 17.0 (SPSS Inc, Chicago, IL, USA). For the description of patients’ characteristics at baseline, mean ± standard deviation (SD) was used for continuous variables and counts with percentages for categorical variables. Shapiro-Wilk test was used to check the normality of variables. For longitudinal comparisons of BCVA and CST between baseline and each time point, the Wilcoxon matched-pairs signed-ranks test was used, since there was deviation from normality; given that four comparisons were done, the level of statistical significance was set at 0.05/4 = 0.012, according to the Bonferroni correction. A p value <0.05 was considered statistically significant, apart from cases where the Bonferroni correction was adopted, as declared above^40^.
